# Evaluating the effect of renin-angiotension-aldosterone system inhibitors and beta blockers on the progression of bicuspid aortic valve aortopathy in adulthood: A retrospective cohort study

**DOI:** 10.1016/j.ijcchd.2026.100653

**Published:** 2026-01-12

**Authors:** Nili Schamroth Pravda, Elchanan Samuel, Tamir Bental, Ofir Brem, Ilan Richter, Keren Skalsky, Miri Schamroth Pravda, Yaron Shapira, Ran Kornowski, Guy Witberg

**Affiliations:** a Adult Congenital Heart Disease Service, Department of Cardiology, Tel Aviv Medical Center, Tel Aviv, Israel; b The Faculty of Medicine, Tel Aviv University, Tel Aviv, Israel; c Department of Cardiology Rabin Medical Center, Petach Tikva, Israel; d David Geffen School of Medicine at UCLA, Los Angeles, CA, USA; e Department of Pediatrics, Mattel Children's Hospital, University of California-Los Angeles, USA; f Internal Medicine H, Edith Wolfson Medical Center, Holon, Israel

**Keywords:** Bicuspid aortic valve, Aortopathy, Renin-angiotensin-aldosterone system inhibitors, Beta blockers

## Abstract

**Purpose:**

Patients with a bicuspid aortic valve (BAV) often have an associated aortopathy and increased risk of aortic dissection. This study aimed to assess if the chronic use of renin-angiotensin-aldosterone system inhibitors (RAASi) or beta blockers (BB) may be associated with reducing the progression of aortic dilation in adult BAV patients.

**Methods:**

A retrospective cohort study was performed including adult patients with BAV with serial echocardiography over ≥5 years. The cohort was subdivided according to chronic use of RAASi or BB. The primary outcome was a composite of progression of absolute ascending aortic diameter ≥4.5 cm or surgery for ascending aortic replacement.

**Results:**

Included were 262 patients with mean age of 54.3 ± 19.5 years and 75.1 % male. The average follow-up time was 10.8 ± 0.8 years. The cumulative incidence of the primary endpoint was 14.6 % at 10-year follow up and 33.3 % at 15-year follow up. While those with RAASi (n = 39) had the primary endpoint numerically less that those without RAASi chronic therapy (12.8 % vs 24.8 %, p = 0.147), on multivariate analysis there was no significant decreased risk for the primary outcome. (HR = 0.89, 95 % CI [0.34–2.86], p = 0.97). Similarly, there was no significant decreased risk of the primary outcome amongst those with chronic beta blocker use following multivariate analysis (HR = 0.96, 95 % CI [0.37–2.51], p = 0.95).

**Conclusions:**

Our findings suggest that the chronic use of RAASi or BB was not associated with a blunted progression of BAV aortopathy in the adult population. These results highlight the need for larger, randomised studies to validate these observations and further explore potential preventative strategies in this population.

## Graphical abstract

Fig. 1: Central Illustration.

**Figure:**
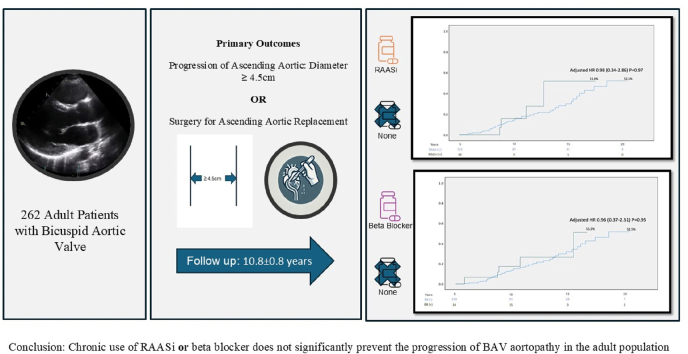

## Teaser

**One-sentence summary:** This study investigates whether medications commonly used for heart conditions, specifically renin-angiotensin-aldosterone system inhibitors (RAASi) and beta blockers, may slow down the progression of aortic enlargement in adults with bicuspid aortic valve (BAV).

**Key findings**

• The use of RAASi and beta blockers was not significantly associated with reducing the progression of aortic dilation in adults with BAV.
• Event rates for the composite endpoint (ascending aortic diameter ≥4.5 cm or aortic surgery) at 10 and 15 years were 14.6 % and 33.3 %, respectively, in this real-world cohort of adult patients with bicuspid aortic valve

## 1 Introduction

Bicuspid aortic valve (BAV) is among the most common congenital cardiac abnormalities, affecting 1.37 % of the global population [[1], [2]]. Patients with a BAV often have an associated aortopathy [3]. Genetic and hemodynamic factors in patients with BAV have been associated with markedly elevated risk of aortopathy progression, and greater than 80 times the risk of aneurysm formation from the general population [1,[4], [5]]. Individuals with BAV-associated aortopathy are at increased risk of aortic dilatation, and in turn, increasing aortic diameters elevate the risk of acute aortic dissection 8-fold, requiring surgical intervention [6]. There is conflicting evidence as to whether renin-angiotensin-aldosterone system inhibitors (RAASi) or beta blockers (BB) can mitigate the growth of the ascending aortic in the paediatric population [7,8]. However, data on this topic in adult populations in limited, and guidelines lack evidence for or against medical treatment of aortic dilatation in the context of BAV [6,9]. This study aims to evaluate the impact of chronic use of RAASi and BB on the progression of aortic dilation in adult BAV patients.

## 2 Methods

We performed a retrospective cohort study including adult (>18 years old) patients with bicuspid aortic valve that had echocardiography performed at our institution between 1996 and 2020. Patients were included if they had at least two echocardiograms separated by a follow-up interval of ≥5 years. Patients with BAV were identified based on formal echocardiography reports documenting bicuspid morphology. Patients who underwent aortic replacement surgery prior to the baseline echocardiogram were excluded.

Baseline characteristics including demographic, clinical, and imaging data was collected retrospectively. Hypertension was defined based on a documented diagnosis in the electronic medical record. Data regarding medication use was taken from prescription data and confirmed with the pharmacy checkout data. Chronic use of medication was defined if there was recorded pharmacy checkout data of the medication during the first as well as the fifth year of follow up. RAASi and beta-blockers were prescribed for clinical indications other than BAV aortopathy (predominantly arterial hypertension). No patient received pharmacotherapy solely for aortic dilation. Ascending aortic and root diameters were extracted from echocardiography reports and measured using standard parasternal long-axis 2-dimensional leading-edge-to-leading-edge systolic dimensions.

The cohort was subdivided according to use of renin-angiotensin-aldosterone system inhibitors (RAASi), beta blockers use or none. The primary outcome was a composite of progression of absolute ascending aortic diameter ≥4.5 cm or surgery for ascending aortic replacement during follow up. A cutoff of 4.5 cm was chosen in accordance with the European Society of Cardiology valvular guidelines IIA indication for replacement of the aortic root or ascending aorta in patients undergoing primary surgery for aortic valve disease [[10], [11]].

### 2.1 Statistics

The cohort was divided into three groups based on the use of the above medications. Baseline characteristics were compared between the groups using the independent sample *t*-test, Wilcoxon sum-rank test and the Chi square test, as appropriate. The cumulative incidence of primary endpoint was assessed using Kaplan-Meier curves and compared between the groups using the Log rank test (unadjusted analysis). Multivariate cox regression analysis was adjusted for age, hypertension, baseline ascending aortic diameter and RAASi/beta blocker usage. Adjusted time to event analysis was performed using a Cox proportional hazards ratio model yielding hazard ratio for the primary endpoint for patients treated/not treated with RAAS-I or beta blockers. Since there were no patients that experienced a clinical event during the first 5 years of follow up, the KM curves were plotted starting at 5 years’ follow up. This project was approved by the local institutional Helsinki Committee.

## 3 Results

**Fig. 1:**
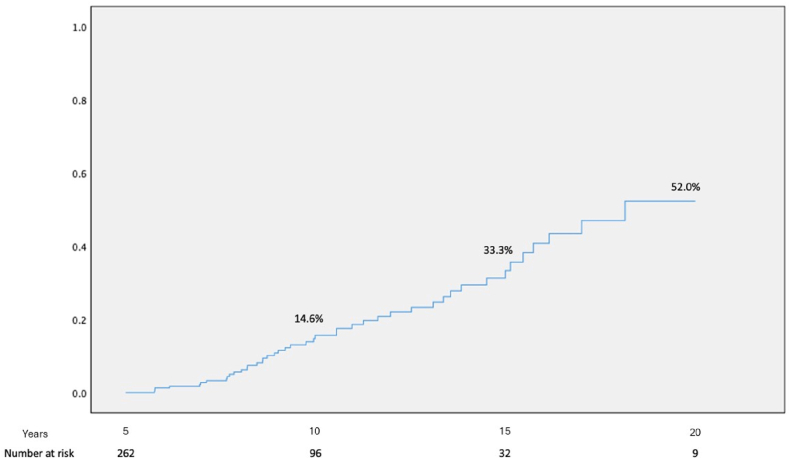
Kaplan Meier Curve for incidence of primary endpoint over follow up time in the cohort.

Included were 262 patients with mean age of 54.3 ± 19.5 years, 75.1 % male. At baseline, the average size of the ascending aorta was 4.0 ± 0.3 cm and 3.4 ± 0.3 cm at the level of the aortic root. The average follow-up time was 10.8 ± 0.8 years. The cumulative incidence of the primary endpoint was 14.6 % at 10-year follow up and 33.3 % at 15-year follow up (Fig. 1).

During follow up, 2 patients died; one patient due to heart failure and the second patient of an unreported cause of death. 23 patients underwent ascending aorta replacement with aortic valve replacement, and 66 patients had progression of ascending aortic diameter ≥4.5 cm. In those undergoing surgery, the primary indication was valvular disease (87 %) with ascending aorta dilation (85 % aortic stenosis, 7.5 % aortic regurgitation and 7.5 % mixed valvular disease).

### 3.1 RAAS-I subgroup

39 patients (15.3 %) were taking RAAS-I. Those taking RAAS-I were significantly older (54.8 ± 13.1 years vs 44.3 ± 14.9 years, p < 0.001), more likely to have hypertension (46.2 % vs 26.0 %, p = 0.013) and more likely to be using concomitant beta blocker therapy (35.9 % vs 8.6 %, p < 0.001)(Table 1). There was no significant difference in the prevalence of ≥moderate aortic stenosis or aortic regurgitation between the subgroups.

**Table 1:** Baseline characteristics according to RAASi usage.

	RAAS-I(+) (n = 39)	RAAS-I(−) (n = 223)	P value
Age	54.8 ± 13.1	44.3 ± 14.9	<0.001
Age<40	8(20.5 %)	97(43.5 %)	0.008
Male	33(84.6 %)	164 (73.5 %)	0.163
Coronary artery disease	10(25.6 %)	31(13.9 %)	0.091
Hypertension	18(46.2 %)	58(26.0 %)	0.013
Dyslipidaemia	18(46.2 %)	73(32.7 %)	0.144
Diabetes	8(20.5 %)	36(16.1 %)	0.491
Coarctation of the aorta	0(0 %)	8(3.6 %)	0.610
Beta blockers	14(35.9 %)	19(8.6 %)	<0.001
Ejection fraction (%)	57.4 ± 6.9	56.6 ± 12.2	0.822
≥Moderate Aortic Stenosis	4 (10.2 %)	27(12.1 %)	0.990
≥Moderate Aortic Regurgitation	21(53.8 %)	70(31.4 %)	0.049
Ascending aorta diameter (cm)	3.9.±0.5	3.9 ± 0.9	0.865
Aortic root diameter (cm)	3.4.±0.7	3.3 ± 0.6	0.838

RAASi = Renin-angiotensin-aldosterone systemic inhibitor.

**Fig. 2:**
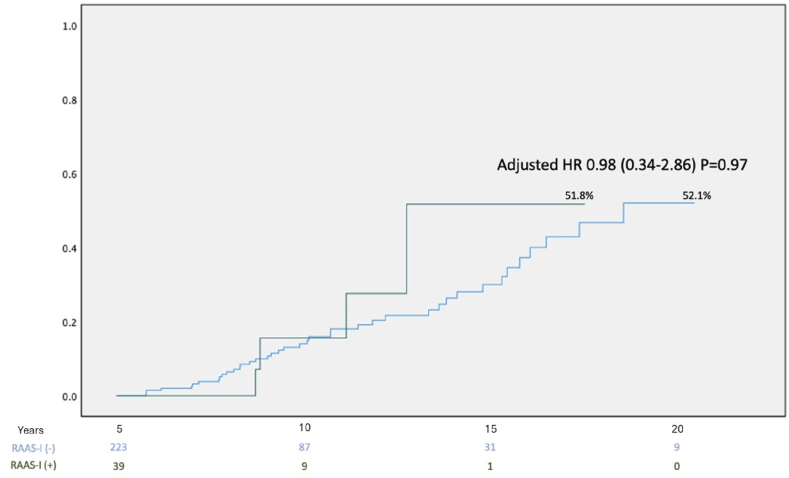
Kaplan Meier Curve for incidence of primary endpoint over follow up time in the cohort according to RAASi chronic usage.

Those with RAAS-I had a non-significant trend to fewer primary endpoint events than those without RAAS-I chronic therapy (12.8 % vs 24.8 %, p = 0.147). On multivariate analysis there was no significant change in primary outcome amongst those with RAASi chronic therapy (HR = 0.89, 95 % CI [0.34–2.86], p = 0.97) as shown in Fig. 2.

### 3.2 Beta blocker subgroup

34 patients (13.0 %) were taking beta blockers. Those taking beta blockers were significantly older (52.4 ± 14.3 years vs 44.8 ± 15.0 years, p = 0.003) and more likely to be using concomitant RAASi (42.4 % vs 11.0 %, p < 0.001). (Table 2).

**Table 2:** Baseline characteristics according to beta blocker usage.

	BB(+) (n = 34)	BB(−) (n = 228)	P value
Age	52.4 ± 14.3	44.8 ± 15.0	0.003
Age<40	8(24.2 %)	97(42.5 %)	0.057
Male	23(69.7 %)	173 (66.3 %)	0.518
Coronary artery disease	10(30.3 %)	31(13.6 %)	0.081
Hypertension	13(39.4 %)	63(27.6 %)	0.217
Dyslipidaemia	15(45.5 %)	76(33.3 %)	0.177
Diabetes	7(21.2 %)	37(16.2 %)	0.461
Coarctation of the aorta	3(9.1 %)	5(2.2 %)	0.067
RAASi	14(42.4 %)	25(11.0 %)	<0.001
≥Moderate Aortic Stenosis	3 (8.8 %)	28 (12.2 %)	0.777
≥Moderate Aortic Regurgitation	11 (32.4 %)	80 (35.1 %)	0.571
Ejection fraction (%)	57.2 ± 7.1	56.9 ± 11.8	0.856
Ascending aorta diameter (cm)	3.8.±0.8	3.9 ± 0.7	0.821
Aortic root diameter (cm)	3.4.±0.8	3.3 ± 0.4	0.842

BB = beta blocker, RAASi = Renin-angiotensin-aldosterone systemic inhibitor.

**Fig. 3:**
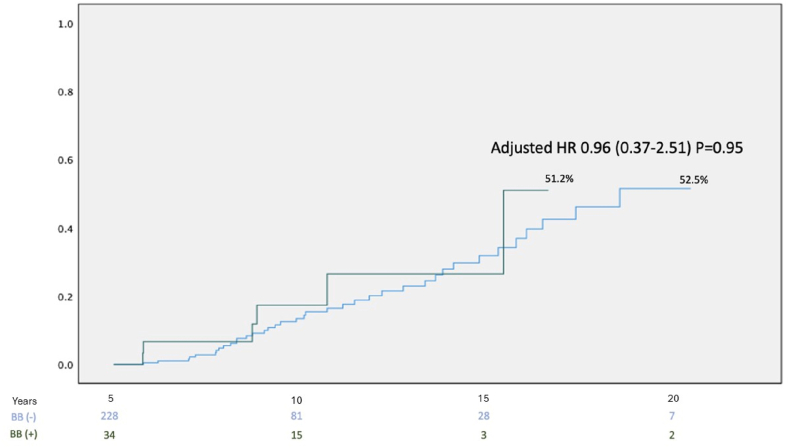
Kaplan Meier Curve for incidence of primary endpoint over follow up time in the cohort according to beta blocker chronic usage.

There was no significant difference in the prevalence of ≥moderate aortic stenosis or aortic regurgitation between the subgroups. On multivariate analysis, there was no significant decreased risk for the primary outcome amongst those with beta blocker chronic therapy (HR = 0.96, 95 % CI [0.37–2.51], p = 0.95) as shown in Fig. 3.

## 4 Discussion

The main findings of our study are that chronic use of RAASi and beta blocker therapy was not significantly associated with reduced risk of aortic dilation ≥4.5 cm or surgery amongst patients with BAV aortopathy.

The development of BAV aortopathy is influenced by multiple factors, including genetics, aortic biomechanics, and hemodynamic factors [4]. RAASi and beta blocker therapy are cardioprotective medications with various mechanisms of action including afterload reduction. Investigating afterload-reducing medication in this disease state is warranted for several reasons. Firstly, arterial hypertension is a known risk factor for the progression of BAV aortopathy, and antihypertensive medications can mitigate this risk [12]. Secondly, afterload reduction may decrease wall stress in the ascending aorta and thus prevent progressive aortic dilation. Thirdly, these medications have shown potential in stabilizing aortic growth in other conditions, such as Marfan-associated aortopathy [[13], [14], [15], [16]]. Our study is not the first to investigate RAAS inhibitors and beta-blockers in BAV aortopathy. However, most prior studies have focused on the paediatric population, where the aorta undergoes dynamic somatic growth and there have been discrepancies in the reported findings [7,8,16]. In adults, there is no somatic growth of the aorta, and aortic growth is pathological, and thus the impact of medications on a stable vascular structure may differ. Our findings showed that both RAAS inhibitor and beta blocker use was not significantly associated with a lower rate of the primary outcome following multivariate analysis. Our findings are similar to a prior imaging study by Allen et al. who investigated the effect of beta blocker usage on aortic blood flow using 4D Magnetic Resonance Imaging [9].They found no significant difference in peak velocity or systolic wall shear stress between those using or not using beta blocker therapy. However, it is interesting to note that our findings did not show a significant difference in hazard ratio despite those in the RAAS inhibitor subgroup being significantly older and with a higher prevalence of hypertension, both of which are risk factors for aortic growth. One might expect patients with older age and hypertension to demonstrate more advanced aortic growth, yet this was not observed in our cohort. Although this finding should be interpreted cautiously, it raises the possibility that medical therapy may have attenuated expected disease progression, or alternatively, that baseline risk factors alone do not fully determine aortic dilation trajectory in BAV. An important caveat to our study is that these patients were prescribed medications for indications other than bicuspid aortopathy and our early findings should be interpreted with caution. These medications are widely available, with few side effects and may play a role in preventing a devastating outcome and thus further large-scale, longitudinal, randomised studies are needed to confirm these findings in the adult population. Given the limited cohort size and low event rates, the study is likely underpowered to detect small-to-moderate treatment effects, and therefore a lack of statistical association should be interpreted with caution. Furthermore, our study was an observational retrospective study with associated biases. We did not have data on the exact dosages of medications administered, family history, or root phenotype. There was overlap between medication subgroups, as a proportion of patients were receiving both RAASi and beta blockers. Additionally, the medication compliance data is limited to pharmacy check out data and assumes adherence to chronic therapy. For these reasons, our retrospective analysis is unable to isolate the effect of each drug class for independent comparative analysis. Echocardiographic data taken was based on a 2-dimensional measurements and our findings should be validated in further studies using 3-dimensional imaging such as cardiac tomography or magnetic resonance imaging that are more precise.

In conclusion, our findings indicate that, while the mechanism of action of RAAS inhibitors and beta-blockers theoretically could prevent the progression of BAV aortopathy in adults, our findings do not support a statistically significant association between RAASi/BB therapy and reduced progression of BAV aortopathy.

## CRediT authorship contribution statement

**Nili Schamroth Pravda:** Writing – review & editing, Writing – original draft, Supervision, Formal analysis, Conceptualization. **Elchanan Samuel:** Investigation, Data curation, Conceptualization. **Tamir Bental:** Resources, Investigation, Conceptualization. **Ofir Brem:** Writing – review & editing, Writing – original draft, Visualization, Resources, Project administration, Investigation, Data curation, Conceptualization. **Ilan Richter:** Investigation, Formal analysis. **Keren Skalsky:** Supervision, Resources, Project administration. **Miri Schamroth Pravda:** Writing – review & editing, Visualization, Validation, Methodology. **Yaron Shapira:** Visualization, Validation, Supervision. **Ran Kornowski:** Writing – review & editing, Validation, Supervision, Investigation, Conceptualization. **Guy Witberg:** Writing – review & editing, Supervision, Resources, Project administration.

## Ethics approval

This project was approved by the local institutional Helsinki Committee. (RMC 955-20)

## Funding

None.

## Declaration of competing interest

The authors declare that they have no known competing financial interests or personal relationships that could have appeared to influence the work reported in this paper.
